# Effectiveness and safety of Guipi Mixture for the treatment of premature ventricular contractions (heart-spleen deficiency syndrome): study protocol for a randomized, double-blind, placebo-controlled, multicentre trial

**DOI:** 10.3389/fcvm.2026.1732874

**Published:** 2026-06-11

**Authors:** Yonghao Li, Yutong Wang, Ruikang Liu, Xuanchun Huang, Shiyi Tao, FuYuan Zhang, Jun Li, JingRun Cui

**Affiliations:** 1 Department of Cardiology, Guang'anmen Hospital, China Academy of Chinese Medical Sciences, Beijing, China; 2 Graduate School, Beijing University of Chinese Medicine, Beijing, China

**Keywords:** efficacy, guipi mixture, premature ventricular contractions, protocol, randomized controlled trial

## Abstract

**Introduction:**

Premature ventricular contractions (PVCs) are a common arrhythmia. Long-term frequent PVCs may contribute to tachycardia-induced cardiomyopathy, exacerbate angina, or trigger more severe ventricular arrhythmias, increasing cardiovascular risk. Current Western guidelines recommend pharmacotherapy or catheter ablation, with conservative drug therapy (e.g., lidocaine, propafenone, metoprolol, amiodarone, verapamil) remaining the mainstay. Metoprolol tartrate, a first-line drug, is effective for PVCs but has single targets, limited efficacy, and potential long-term cardiovascular adverse effects. Traditional Chinese Medicine (TCM) offers high efficacy and low toxicity. Guipi Mixture, a Chinese patent medicine for heart-spleen deficiency syndrome, combined with Western medicine may overcome these limitations. This study evaluates the efficacy and safety of Guipi Mixture plus metoprolol for PVCs (heart-spleen deficiency syndrome).

**Methods:**

This is a randomized, double-blind, placebo-controlled, multicenter trial. Across 18 centers, 200 eligible subjects will be randomized (1:1) to intervention (Guipi Mixture + metoprolol) or control (Guipi Mixture placebo + metoprolol). The 12-week trial includes a 2-week screening, 2-week run-in, and 8-week treatment period. The primary outcome is the absolute change in 24 h PVC count from baseline to week 8 measured by ambulatory ECG (AECG). Secondary outcomes include responder rate (based on PVC reduction), SF-36 quality of life, PSQI sleep quality, TCM syndrome score, and ECG indicators. Adverse events will be recorded throughout the trial.

**Discussion:**

This study will provide evidence on the efficacy and safety of Guipi Mixture for PVCs (heart-spleen deficiency syndrome) and explore its impact on patients’ quality of life.

**Clinical Trial Registration:**

http://itmctr.ccebtcm.org.cn/, identifier ITMCTR2025001333.

## 1 Introduction

Arrhythmia is a common cardiovascular disease with an acute onset, often complicated by other cardiovascular diseases. If not treated promptly, it can impair cardiac function and even lead to sudden death, seriously threatening patients' life and health (1, 2). Ventricular arrhythmias are very common clinically and mainly include premature ventricular contractions (PVCs), ventricular tachycardia, ventricular flutter, and ventricular fibrillation. PVCs are a relatively common manifestation of the heart; even individuals with normal hearts may experience them due to stimulation or medication (3). The incidence of PVCs in the general population is 1%–4% (4). The incidence of PVCs increases with age. Some patients experience severe anxiety, insomnia, and other discomforts due to frequent PVCs, forming a vicious cycle that makes PVCs more frequent, affecting patients' daily life and work. Long-term PVCs can easily lead to heart failure, angina pectoris, arrhythmia, and in severe cases, even endanger life. Therefore, the treatment of PVCs is very important (5).

Current Western clinical guidelines recommend pharmacological therapy and catheter ablation for treating PVCs (6, 7). Catheter ablation is suitable for patients with symptomatic frequent PVCs, but it is an invasive treatment with poor patient compliance. Currently, conservative drug therapy remains the mainstay clinically (8), including lidocaine, propafenone, metoprolol, amiodarone, verapamil, etc. Among these, metoprolol tartrate tablets, as a first-line drug, have a positive effect on PVCs. Metoprolol tartrate tablets are β-receptor blocking agents. By inhibiting cardiac β-adrenergic receptors, they block excitatory conduction, reduce myocardial oxygen consumption, and achieve the goal of slowing the heart rate (9). However, Western medicine therapy has relatively single therapeutic targets, limited efficacy, and long-term use can easily cause certain adverse effects on patients' cardiovascular function (10).

TCM offers advantages of high efficacy and low toxicity in treating PVCs, providing new strategies for treating this disease. PVCs belong to the category of “palpitations” (Xinji) in TCM. It is a syndrome of root deficiency and branch excess, with insufficiency of Qi and blood and deficiency of Yin and Yang as the root, and Qi stagnation, blood stasis, and phlegm-fluid retention as the branch. Heart-spleen deficiency syndrome is a common pattern of this disease. TCM treatment emphasizes the holistic concept and pattern differentiation-based treatment, aiming to treat the disease by regulating the balance of Yin and Yang, supporting the healthy Qi and eliminating pathogens, and addressing the root cause. Guipi Mixture is a Chinese patent medicine preparation with the effects of tonifying Qi, fortifying the spleen, nourishing blood, and calming the spirit. It is commonly used clinically to treat heart-spleen deficiency syndrome. Previous studies (11) have shown that combined Chinese and Western medicine can be used for the clinical treatment of arrhythmia of the heart-spleen deficiency type. Compared with Western medicine treatment alone, Guipi Mixture combined with Western medicine can significantly improve the total effective rate (*P* < 0.05), significantly alleviate symptoms such as palpitations, shortness of breath, dizziness and vertigo, and further improve patients’ clinical symptoms and cardiac function, enhancing the therapeutic effect. (The drug components of Guipi Mixture are detailed in Table 1).

**Table 1:** The composition of Guipi Mixture (intervention drug).

Chinese name	Chinese pinyin	English name	Latin name	Plant family	Part used	Daily dosage (g)
党参	Dang Shen	Codonopsis Root	*Codonopsis pilosula*	Campanulaceae	Root	12
白术	Bai Zhu	Atractylodes Rhizome	*Atractylodes macrocephala*	Asteraceae	Rhizome (stir-fried with bran)	9
黄芪	Huang Qi	Astragalus Root	*Astragalus membranaceus*	Fabaceae	Root (honey-processed)	12
甘草	Gan Cao	Licorice Root	*Glycyrrhiza uralensis*	Fabaceae	Root (honey-processed)	6
茯苓	Fu Ling	Poria Fungus	*Wolfiporia extensa*	Polyporaceae	Sclerotium	10
远志	Yuan Zhi	Polygala Root	*Polygala tenuifolia*	Polygalaceae	Root (processed with licorice)	6
酸枣仁	Suan Zao Ren	Spina Date Seed	*Ziziphus jujuba var. spinosa*	Rhamnaceae	Seed (fried)	12
龙眼肉	Long Yan Rou	Longan Aril	*Dimocarpus longan*	Sapindaceae	Aril (fruit flesh)	12
当归	Dang Gui	Chinese Angelica Root	*Angelica sinensis*	Apiaceae	Root	9
木香	Mu Xiang	Costus Root	*Aucklandia costus*	Asteraceae	Root	6
大枣	Da Zao	Jujube Fruit	*Ziziphus jujuba*	Rhamnaceae	Fruit (seed removed)	20
生姜	Sheng Jiang	Fresh Ginger	*Zingiber officinale*	Zingiberaceae	Rhizome	10

Given the current treatment status of PVCs and previous data on Guipi Mixture, this study aims to further evaluate the efficacy and safety of Guipi Mixture in treating PVCs (heart-spleen deficiency syndrome).

## 2 Methods and analysis

### 2.1 Study design

This trial is led by the Guang'anmen Hospital, China Academy of Chinese Medical Sciences, and will be conducted in 18 hospitals across China (Guang'anmen Hospital, China Academy of Chinese Medical Sciences; Fengxian People's Hospital; Zigong Fourth People's Hospital; Yilong County People's Hospital; Yiyuan County People's Hospital; Xiangtan First People's Hospital; General Hospital of Hunan University of Medicine; Karamay Central Hospital; Affiliated Hospital of Jiangsu University; Taizhou People's Hospital; Wuzhou Workers' Hospital; Xiangtan County People's Hospital; Liuyang Traditional Chinese Medicine Hospital, Changsha; Affiliated Hospital of Jiujiang University; The Fourth Affiliated Hospital of Nanchang University; Affiliated Hospital of Jinggangshan University; Yichun People's Hospital; Xining First People's Hospital).

**Figure 1:**
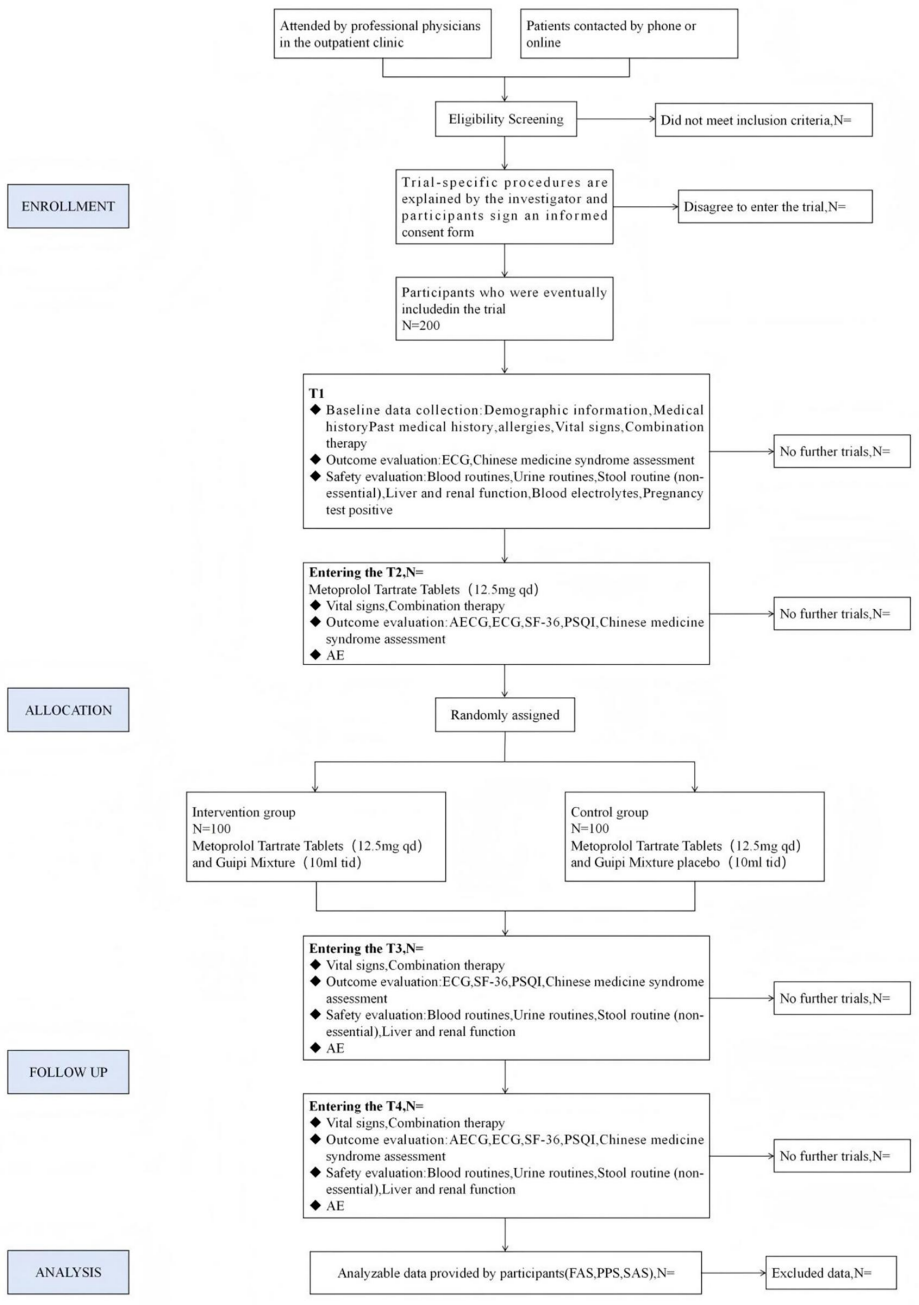
The flow diagram of the trial.T1, screening period 2 weeks, -28-14 days; T2, Introduction period 2 weeks, -14-0 days; T3, Introduction period 2 weeks, week 4 ± 3 days; T4, intervention period 8 weeks, week 8 ± 3 days; AECG, ambulatory electrocardiogram; ECG, electrocardiogram; CRF, case report form; SF-36, short form 36 health survey; PSQI, Pittsburgh sleep quality index; AE, adverse event.

This study adopts a randomized, double-blind, placebo-controlled research design, with a 1:1 allocation ratio, using Guipi Mixture combined with metoprolol tartrate tablets to treat PVCs (heart-spleen deficiency syndrome) (Figure 1). Evaluations and visits will be conducted according to the detection schedule (Table 2). This study will restrict subject enrollment, use strict randomization and blinding, and apply statistical methods to prevent potential biases inherent in these approaches.After the recruitment period and obtaining signed informed consent from all subjects, eligible subjects will be randomly assigned to the experimental group or the control group and receive 8 weeks of treatment.

**Table 2:** Time schedule of enrolment, intervention and outcome measures of the trial.

Study phase	Screening period 2 weeks	Introduction period 2 weeks	Intervention period 8 weeks		Early departure from the group/Termination of the trial
Time point	T1	T2	T3	T4	
	-28-14 days	-14-0 days	Week 4 ±3 days	Week 8 ±3 days	
Face-to-face meeting Enrolment	×	×	×	×	×
Eligibility screening Inclusion/Exclusion Criteria examination	×				
Informed consent	×				
Allocation	×				
Baseline data collection					
Demographic information	×				
Medical history	×				
Past medical history, allergies	×				
Vital signs	×	×	×	×	×
Combination therapy	×	×	×	×	×
Outcome evaluation					
AECG		×		×	×
ECG	×	×	×	×	×
SF-36		×	×	×	×
PSQI		×	×	×	×
Chinese medicine syndrome assessment	×	×	×	×	×
Safety evaluation					
Blood routines	×		×	×	×
Urine routines	×		×	×	×
Stool routine (non-essential)	×		×	×	×
Liver and renal function	×		×	×	×
Blood electrolytes	×				
Pregnancy test positive	×				
Investigational Medication Distribution		×			
Investigational Medication Collection				×	×
Medication quantity statistics				×	×
AE		×	×	×	×
Log Cards Issuance	×				
Log Cards Collection				×	×
Schedule a follow-up visit	×	×	×		
CRF monitor examination				×	
CRF principal investigator examination				×	

T1, Screening period 2 weeks, -28-14 days; T2, Introduction period 2 weeks, -14-0 days; T3, Introduction period 2 weeks, Week 4 ± 3 days; T4, Intervention period 8 weeks, Week 8 ± 3 days; AECG, ambulatory electrocardiogram; ECG, electrocardiogram; CRF, case report form; SF-36, short form 36 health survey; PSQI, pittsburgh sleep quality index; AE, adverse event.

The study was approved by the Ethics Committee of Guang'anmen Hospital, China Academy of Chinese Medical Sciences (approval number: 2024-115-KY-01) and registered at the International Traditional Medicine Clinical Trial Registry (http://itmctr.ccebtcm.org.cn/) on July 3, 2025 (Registration Number: ITMCTR2025001333). We designed this protocol according to the Standard Protocol Items: Recommendations for Interventional Trials (SPIRIT). The purpose, procedures, and potential side effects of the study will be explained to participants. Signed informed consent will be obtained from all subjects. Participants' names or identity information will not be published.

### 2.2 Sample size calculation

This study is a randomized controlled trial. The primary outcome is the absolute change in 24 h PVC count from baseline to week 8, which will be analyzed as a continuous variable using a superiority test design. Subjects will be randomly assigned to the experimental group (Guipi Mixture + metoprolol) and the control group (Guipi Mixture placebo + metoprolol) in a 1:1 ratio.

Referring to a previous multicenter randomized controlled trial of a Chinese patent medicine combined with metoprolol in patients with frequent PVCs (12), the between-group difference in PVC count reduction was 1,524 beats per 24 h, with a pooled standard deviation (SD) of approximately 3,200. This yields an effect size (Cohen's d) of 0.48 (1,524/3,200). Assuming a two-sided α = 0.05, β = 0.2 (80% power), and a 1:1 allocation ratio, the required sample size for each group is calculated as 69 subjects using the standard formula for two independent samples. After considering a 20% dropout rate, the final sample size is 83 subjects per group. To ensure sufficient power for prespecified subgroup analyses and exploratory endpoints, we conservatively set the enrollment target at 100 subjects per group (200 subjects in total). According to the plan, Guang'anmen Hospital, China Academy of Chinese Medical Sciences, as the leading center, will complete the enrollment of 20 subjects, and the remaining 18 centers will equally distribute the remaining 180 subjects.

### 2.3 Eligibility criteria

For outpatient or inpatient patients seeking medical attention, if they meet the diagnostic criteria for PVCs in the “2020 Chinese Expert Consensus on Ventricular Arrhythmias (Upgraded from 2016 Version)" (13) and the pattern differentiation criteria for heart-spleen deficiency syndrome in the “Guiding Principles for Clinical Research of New Chinese Medicines" (14) (Tables 3, 4), they will be assessed against the inclusion and exclusion criteria (shown below). Patients who do not meet any inclusion criterion or meet any exclusion criterion will not be recruited. If a patient meets one of the withdrawal or termination criteria, they will be removed from the trial. Participants will be required to provide written informed consent to cooperate with the doctor's treatment and follow-up and to provide information not exceeding the scope of ethical approval. Subjects who do not meet the requirements will be removed from the study. During this period, the subject's medical history, physical examination, blood samples, and medical records will be collected and saved.

**Table 3:** Western diagnostic criteria diagnosis of premature ventricular complexes (PVCs). Referenced:2020 Chinese expert consensus on ventricular arrhythmias (Updated from 2016 version).

Item	Description	
Clinical Manifestations	PVCs exhibit high heterogeneity in symptom presentation. Most patients are asymptomatic, whereas even sporadic PVCs may cause severe symptoms including palpitations, chest tightness, and sensation of skipped beats. In some cases, PVCs can reduce cardiac output and compromise perfusion to vital organs, leading to fatigue, dyspnea, diaphoresis, and dizziness.	
Diagnostic Basis	Resting 12-lead electrocardiogram (ECG) 24-hour ambulatory ECG (Holter monitoring)	
Lown Grading System for PVCs	Grade	Definition
	0	No PVCs
	I	Occasional PVCs (<2/min or <30/h)
	II	Frequent PVCs (≥2/min or ≥30/h)
	III	Multiform PVCs
	IVa	Couplets (paired PVCs)
	IVb	Salvos (≥3 consecutive PVCs)
	V	R-on-T phenomenon

**Table 4:** Traditional Chinese medicine (TCM) diagnostic criteria. Referenced:guiding principles for clinical research of new Chinese medicines (2002).

Item	Description
Core Symptoms (Required)	♦ Palpitations (Xinji) ♦ Shortness of breath (Qiduan)
Accompanying Symptoms (≥2 required)	♦ Dizziness (Xuan Yun) ♦ Blurred vision (Mu Xuan) ♦ Insomnia with dream-disturbed sleep (Shao Mei Duo Meng) ♦ Poor memory (Jian Wang) ♦ Pallor (Mian Se Wu Hua) ♦ Lethargy (Shen Pi Juan Fa) ♦ Anorexia (Na Dai Shi Shao) ♦ Abdominal distension and loose stools (Fu Zhang Bian Tang)
Tongue Presentation	♦ Pale-red tongue (Dan Hong She) ♦ Thin white coating (Bo Bai Tai)
Pulse Presentation	♦ Thready pulse (Xi Mai) ♦ Weak pulse (Ruo Mai) ♦ Deep and thready pulse (Chen Xi Mai) ♦ Thready and weak pulse (Xi Ruo Mai)

Patients must exhibit both core symptoms and at least two accompanying symptoms, with the diagnosis of Heart-Spleen Deficiency Syndrome confirmed through comprehensive pattern differentiation based on the four diagnostic examinations.

#### 2.3.1 Inclusion criteria

Patients must meet all of the following criteria to enter this project:

1. Age ≥18 years and ≤75 years, regardless of gender;
2. Meet the Western diagnostic criteria for functional PVCs, and have ≥720 PVCs/24 h on 24-hour AECG (13);
3. Meet the TCM pattern differentiation criteria for heart-spleen deficiency syndrome;
4. Meet Lown grade II to IVa (as assessed by the grading criteria referenced in the 2020 Chinese Expert Consensus on Ventricular Arrhythmias (13), noting that PVC grading should be interpreted in conjunction with the patient's overall clinical status and underlying cardiac condition).
5. PVC symptoms have persisted for more than 1 week;
6. Provide informed consent and sign the informed consent form.

#### 2.3.2 Exclusion criteria

Patients meeting any of the following criteria will not be allowed to enter this project:

1. Patients with comorbid hypertension whose blood pressure remains unstable after standardized antihypertensive treatment, i.e., systolic blood pressure ≥160 mmHg and/or diastolic blood pressure ≥100 mmHg;
2. Patients with various structural heart diseases such as coronary heart disease, heart failure, cardiomyopathy, valvular heart disease, mitral valve prolapse, pacemaker implantation, etc.; Patients with malignant ventricular arrhythmias (such as short runs of ventricular tachycardia, torsades de pointes, ventricular fibrillation, etc.), prolonged QT syndrome (QT interval ≥500 ms), or severe conduction block (e.g., II, III degree) or other supraventricular arrhythmias; Patients with comorbid malignant tumors, severe cerebrovascular diseases, liver or kidney dysfunction, infectious diseases, hematological or other systemic diseases, or mental illness;
3. Patients whose PVCs are caused by drugs, electrolyte imbalances (hypokalemia, hypomagnesemia), anemia, hyperthyroidism, or other systemic diseases or unknown reasons;
4. Patients with known allergies to any component of the investigational drugs;
5. Serum creatinine exceeding the upper limit of normal or ALT or AST exceeding twice the upper limit of normal;
6. Women who are pregnant, planning pregnancy within six months, or breastfeeding;
7. Patients suspected or confirmed to have alcohol addiction or history of drug abuse;
8. Patients who cannot discontinue other antiarrhythmic drugs besides metoprolol tartrate tablets during this clinical trial phase;
9. Patients with indications for surgery and willingness to undergo surgical treatment;
10. Patients who have participated in other clinical trials within 30 days before screening.

### 2.4 Recruitment

A total of 200 patients will be recruited from the 18 clinical trial institutions participating in this study. Investigators, assisted by trained research coordinators at each participating center, will identify potentially eligible patients based on the eligibility criteria. They will collaborate with the attending physicians to determine if patients are eligible for the trial. Patients seeking outpatient or inpatient treatment for PVCs will be assessed for eligibility by the investigators, who will collect relevant information face-to-face with the patient's verbal consent during treatment. If PVC patients are recruited through posters, the internet, etc., eligibility will be assessed by phone.

### 2.5 Informed consent

After a patient is deemed eligible to participate, the investigator must describe the details of the clinical trial in detail, including the purpose of the trial, trial procedures, potential benefits and risks, the rights and obligations of the subjects, etc., so that the subject fully understands and has sufficient time to consider. After their questions are satisfactorily answered, the subject will sign the informed consent form. When each participant signs the informed consent form, the doctor will leave his or her contact phone number with the participant so that he or she can contact the doctor at any time if circumstances change.

### 2.6 Randomization and blinding

Bias refers to systematic error in the conclusions of a clinical trial compared to the true situation due to the influence of certain factors, which may exaggerate or diminish the authenticity of the results. Bias can occur at any stage from the design to the implementation and finally to data analysis and conclusion derivation in a clinical trial. This trial adopts a randomized, double-blind, placebo-controlled design to avoid bias caused by individual differences.

#### 2.6.1 Randomization

In this study, centralized randomization will be performed using an Interactive Web Response System (IWRS) hosted by an independent Contract Research Organization (CRO). The system will be accessible to all 18 participating centers via a secure web interface. A computer-generated randomization sequence with permuted blocks (block size of 4, randomly varied) will be used, stratified by study center and baseline PVC burden (<10,000/24 h vs. ≥10,000/24 h). Subjects will be randomly assigned to the experimental group or the placebo control group in a 1:1 ratio.

After written informed consent is obtained and eligibility is confirmed, the investigator or an authorized designee will log into the IWRS, enter the subject's screening number and baseline information, and confirm that all inclusion/exclusion criteria are met. The system will then immediately assign the next available randomization number from the central list and reveal the corresponding treatment allocation. Allocation concealment is ensured because the treatment assignment is generated centrally and is only released after the participant is irrevocably enrolled. No site staff can predict the next assignment before completing the enrollment process, and no site-specific randomization lists, sealed envelopes, or other physical allocation tools will be used.

Throughout the trial, investigators must not modify the generated random numbers or the assigned group. If a subject is randomized but withdraws from the trial before receiving any study treatment, the assigned randomization number will be retained and permanently retired (i.e., not reused for any other subject). The randomization code will remain inaccessible to all investigators, participants, and outcome assessors until database lock.

To ensure subject safety, an emergency unblinding procedure is predefined. When a treating investigator needs to know the subject's actual treatment assignment for a medical necessity (e.g., a serious adverse event), an emergency unblinding request can be submitted through the IWRS. The system will log the reason, immediately display the treatment allocation, and automatically archive all unblinding events and their reasons for subsequent submission to the sponsor and regulatory authorities at the end of the trial. To reduce or avoid bias, the quality control regulations of the trial protocol should be strictly followed.

#### 2.6.2 Blinding

The randomization code (blinding code) of the study will be generated by non-blinded biostatistics professionals using statistical software before the formal enrollment of subjects. It will be kept blinded from subjects, investigators, the research team, and other personnel who need to remain blinded until database lock. Before the start of the trial, non-blinded biostatisticians will code the drugs according to the randomization code. The investigational drug and its corresponding placebo simulator must be completely identical in appearance, smell, taste, packaging, labeling, dosing regimen, etc., to maintain confidentiality of the treatment assignment. After drug coding is completed, the randomization blinding code will be sealed and must not be opened during the trial. The blinding code will remain sealed until all outcome assessments are completed. All 24-hour Holter electrocardiogram recordings will be sent to a central core laboratory (Guang'anmen Hospital) for blinded analysis; the Holter readers will be unaware of both treatment allocation (experimental vs. control) and time point (baseline vs. follow-up). Traditional Chinese Medicine (TCM) pattern assessments will be performed by trained TCM practitioners who are also blinded to group assignment. Access to randomization data should be strictly controlled before overall data unblinding to prevent disclosure of the blinding code.

### 2.7 Interventions

According to the study's inclusion/exclusion criteria, eligible subjects will enter a 2-week run-in period to reduce interference from previous medications on the study results and obtain relatively stable and reliable baseline data. All subjects in the run-in period will take metoprolol tartrate tablets. Afterwards, randomization will be performed in a 1:1 ratio. Baseline data collection (corresponding visit) will be completed on the day of randomization, followed by an 8-week double-blind treatment period. During the treatment period, the experimental group will receive Guipi Mixture (10 mL, three times daily) (Lunan Houpu Pharmaceutical Co., Ltd.) (Table 1) added to the baseline treatment of metoprolol tartrate tablets (12.5 mg, once daily) (AstraZeneca Pharmaceuticals LP). The control group will receive Guipi Mixture placebo (10 mL, three times daily) (Lunan Houpu Pharmaceutical Co., Ltd.).

The placebo for Guipi Mixture was formulated by the same manufacturer (Lunan Houpu Pharmaceutical Co., Ltd.) using identical excipients (purified water, sucrose, caramel color) as the active preparation, but without the herbal extract. To match the color, caramel color was used at the same concentration as in the active product. To replicate the characteristic bitter and astringent taste, food-grade bittering agent (e.g., quinine sulfate) and tannic acid were added at predetermined concentrations. The odor was matched by using the same excipient system without additional fragrances, as preliminary assessment showed the excipient mixture alone produced a similar odor profile. The placebo will be identical in appearance, packaging, labeling, and dosing regimen to the active Guipi Mixture. Prior to trial initiation, a pilot sensory similarity evaluation will be conducted with 10 healthy volunteers (not involved in the study) to confirm that the placebo is indistinguishable from the active product in terms of appearance, color, taste, and smell (*P* > 0.05 for all comparisons). This step will ensure the reliability of the double-blind design.

The investigator or their authorized personnel (e.g., pharmacist) is responsible for ensuring that all investigational products are stored in a secure area with controlled access that meets storage conditions and complies with applicable regulatory requirements. The investigator and/or the research site should establish a system for investigational product management to ensure: The use of investigational drugs is the responsibility of the investigator. The investigator must ensure that all investigational drugs are used only for subjects in this clinical trial. Their usage and dosage should follow the trial protocol; research drugs must not be given to any non-clinical trial participant. The investigator should designate a specific person responsible for the storage, dispensing, recovery, and recording of investigational drugs. Records should include quantity, shipment, delivery, receipt, allocation, recovery and destruction of remaining drugs after use, etc. Drug requisition must be handled according to regulations. All drugs must be registered upon receipt, carefully filling out the registration form, including the recipient, checker, date of receipt, dosage and quantity of drugs received. Remaining drugs after subject administration need to be recovered and properly stored after cross-checking with the diary card and/or prescription form, and/or the quantity on the medical order. Remaining drugs and packaging will be uniformly recovered, returned, and destroyed. Subjects will complete relevant visits at weeks 4 and 8 of medication use.

Concomitant medication/treatment refers to other drugs/treatments administered at the discretion of the investigator based on the subject's best interests. All concomitant medications, blood products, and non-drug interventions (e.g., punctures) received by subjects during the study should be strictly recorded in the Case Report Form (CRF) in accordance with Good Clinical Practice (GCP) regulations.

The study protocol explicitly prohibits the use of other antiarrhythmic drugs, and other TCMs, decoctions, or formula granules for tonifying Qi and nourishing blood, such as Buzhong Yiqi Wan, Sijunzi Wan, Siwu Tang, Bazhen Wan, Shiquan Dabu Wan, etc., throughout the entire study period.

If a subject develops a comorbid condition that necessitates the use of prohibited medications, it may be necessary to discontinue the investigational drug treatment or administer the prohibited drug. The investigator will discuss whether the subject should continue to participate in the study treatment.

Participants will take the medication daily for 8 consecutive weeks under self-monitoring. During the drug intervention, we will strictly ensure subject compliance. After a subject formally decides to participate in the clinical study, the investigator will explain the entire trial process, including the time points for collecting various types of information, the meaning of each term in the questionnaires, and the frequency and method by which the subject needs to take the medication. Subjects will need to fill out a daily health monitoring form, recording drug intake and changes in their condition for the investigator to check. Participants will be asked to bring all unused medication to each follow-up visit to check their compliance. The investigator will accurately record the quantity of drugs received, administered, and returned by the subject and promptly document it in the CRF. Once a subject is enrolled in the study, the drugs used and the examinations involved will be covered by the research center. Upon completion of the trial, subjects will receive a certain amount of grant money. Through these incentives, participants will maximize their enthusiasm for the trial, which is expected to improve the clinical condition of PVCs.

The fixed dose of metoprolol tartrate (12.5 mg once daily) was selected based on the standard clinical practice in Chinese community hospitals and primary care settings for initial management of symptomatic PVCs (15). To ensure safety, Participants with baseline heart rate < 60 bpm or systolic blood pressure < 100 mmHg will be excluded. During the trial, if a participant develops symptomatic bradycardia (heart rate < 50 bpm with dizziness/ fatigue) or hypotension (systolic BP < 90 mmHg), the dose may be reduced to 6.25 mg once daily or temporarily discontinued. Dose up-titration (to 12.5 mg twice daily) is permitted at week 4 only if heart rate remains > 80 bpm and blood pressure is stable. Such dose modifications will be documented as protocol deviations. Primary analysis will be by intention-to-treat (ITT); a per-protocol sensitivity analysis excluding patients with major dose modifications will also be performed.

Furthermore, the 2020 Chinese expert consensus on ventricular arrhythmias (13) emphasizes that β-blockers should be initiated at a low dose and gradually up-titrated according to the patient's tolerance. This principle aligns with the low-dose initiation and flexible adjustment design of this trial.

### 2.8 Outcome evaluation

#### 2.8.1 Primary outcome

The primary efficacy outcome is the absolute change in the total number of PVCs per 24 h from baseline (end of run-in period, T2) to week 8 (end of treatment, T4), measured by 24 h AECG. If the data are highly skewed, a log-transformed change or percent change will be analyzed as a sensitivity measure.

#### 2.8.2 Secondary Outcomes

1. Change from baseline in the clinical efficacy of subjects in both groups after 8 weeks of treatment (13). Based on the following clinical efficacy evaluation criteria, the total effective rate (Total effective rate = Markedly effective rate + Effective rate) and the difference between the two groups will be calculated.
  ♦ Markedly Effective: Clinical symptoms such as palpitations and shortness of breath disappear significantly; 24 h AECG shows a reduction in PVC count ≥90%.
  ♦ Effective: Clinical symptoms such as palpitations and shortness of breath improve; 24 h AECG shows a reduction in PVC count between 50% and 90%.
  ♦ Ineffective: Clinical symptoms such as palpitations and shortness of breath show no improvement or even worsen; 24 h AECG shows a reduction in PVC count <50% or an increase.
2. Change from baseline in Quality of Life (SF-36) score at 4 and 8 weeks of treatment (16).
3. Change from baseline in Pittsburgh Sleep Quality Index (PSQI) score at 4 and 8 weeks of treatment (17).
4. Change from baseline in TCM syndrome total score and individual symptom scores (14).
5. Change from baseline in ECG indicators (18) after 8 weeks of treatment. The measured values of each ECG indicator will be recorded in detail for efficacy analysis.

#### 2.8.3 Safety outcomes

Safety evaluation indicators mainly include vital signs, physical examination, blood routine, urine routine, stool routine, liver function, kidney function, 12-lead ECG, 24 h AECG, and clinical symptoms. Any adverse events occurring during the study period for all subjects, including abnormalities in clinical symptoms and vital signs, and abnormalities in laboratory tests, will be recorded, noting their clinical manifestations, severity, time of occurrence, duration, management methods, prognosis, and their relationship to the investigational drug. The safety of the investigational drug will be evaluated using the National Cancer Institute Common Terminology Criteria for Adverse Events (NCI CTCAE) version 5.0. The collection period for serious adverse events (SAEs) should start from the subject signing the informed consent form until the end of patient follow-up. If an SAE occurs, the clinician must immediately fill out the “Serious Adverse Event Report Form” within 24 h of becoming aware, sign and date it, and immediately report it to the hospital ethics office and the drug safety department of the trial drug manufacturer. The investigator should decide whether to stop the trial based on the severity. Such samples will be considered non-cases. The research team will continue to track the progress of adverse events.

Recent literature highlights that drug-related adverse events are more frequent in patients with polypharmacy, advanced age, and underlying cardiac conditions (19, 20). Although our study enrolls adults without severe structural heart disease, we recognize that the combination of Guipi Mixture (multiple herbal components) and metoprolol may pose additional risks. Therefore, we will implement rigorous adverse event monitoring using NCI CTCAE v5.0, and any signal of hepatotoxicity, nephrotoxicity, or proarrhythmia will prompt immediate unblinding and discontinuation. These measures are now explicitly stated.

#### 2.8.4 Exploratory analysis indicators

Baseline data of PVCs will be recorded in detail, including Lown grade, baseline PVC count, disease duration, comorbidities, ECG indicators, etc., to explore the impact of different Lown grades on efficacy and the characteristics of the population benefiting most from Guipi Mixture for PVCs; Data from Chinese and Western medicine efficacy evaluations before and after treatment will be recorded in detail to explore the correlation between TCM syndrome and PVC count assessment.

### 2.9 Definition and selection of analysis sets

#### 2.9.1 Full analysis set (FAS)

According to the Intention-to-Treat (ITT) principle, all randomized subjects with at least one medication record and one efficacy evaluation will be included in the FAS. The FAS is the primary population for efficacy evaluation in this study.

#### 2.9.2 Per-protocol set (PPS)

All subjects who completed the treatment specified in the protocol or did not have major protocol violations. The exact definition of major protocol violation will be finalized during data review and may generally include (but is not limited to) the following situations: failure to meet major inclusion criteria, treatments received after enrollment that seriously interfere with efficacy evaluation, poor compliance, follow-up seriously exceeding the time window, etc. The PPS is the secondary population for efficacy analysis.

#### 2.9.3 Safety analysis set (SAS)

Subjects who received at least one dose of the study medication and have safety evaluation data.

### 2.10 Statistical analysis methods

All statistical calculations will be performed using SAS 9.4 or higher version statistical analysis system programming. Hypothesis testing generally uses two-sided tests with a significance level of α = 0.05 (unless otherwise specified), providing the test statistic and its corresponding two-sided *P*-value. When Fisher's exact probability method is used, the *P*-value will be given directly. *P* ≤ 0.05 will be considered statistically significant for the tested difference. Confidence intervals will use a 95% confidence level.

#### 2.10.1 General analysis

All statistical analyses for this project will be completed using SAS 9.4 (or higher). Unless otherwise specified, all statistical tests will be two-sided, and *P* < 0.05 (two-sided) will be considered statistically significant. Measurement data will be described using number of cases, mean, standard deviation (STD), median, minimum and maximum values. Count data will be summarized using frequency and percentage. For comparing baseline characteristics between groups: comparison of quantitative indicators between groups will use group t-test or Wilcoxon rank-sum test based on data distribution; categorical indicators will use chi-square test or Fisher's exact probability method; ordinal data will use Wilcoxon rank-sum test.

#### 2.10.2 General information and baseline characteristics

Analysis of quantitative data will describe the number of cases, mean, standard deviation, minimum, median, maximum, upper quartile (Q1), lower quartile (Q3), 95% confidence interval (95% CI), and use t-tests for between-group comparisons. Analysis of qualitative data will describe the number of cases and their percentage, and use chi-square test or Fisher's exact probability method for between-group comparisons. Baseline comparisons include: demographic characteristics, vital signs, physical examination, disease-related factors, and history of other disease medications.

#### 2.10.3 Efficacy analysis

Efficacy analyses will be performed using both the Full Analysis Set (FAS) and the Per-Protocol Set (PPS), with FAS serving as the primary analysis population.

- **Primary Efficacy Outcome:** The primary outcome is the absolute change in 24 h PVC count from baseline (end of run-in, T2) to week 8 (end of treatment, T4). Given that PVC count data are typically right-skewed, the change will be analyzed after log-transformation (log[post-treatment PVC count + 1]−log[baseline PVC count + 1]). A mixed-effects model for repeated measures (MMRM) will be used, including fixed effects for treatment group, visit (week 4 and week 8), treatment-by-visit interaction, and baseline PVC count as a covariate. Site will be included as a random effect. The primary comparison of interest is the difference between the two treatment groups in the change from baseline at week 8. Least squares means (LSMs) and their 95% confidence intervals will be reported. As a secondary approach, an analysis of covariance (ANCOVA) will also be performed with baseline PVC count as a covariate.
- **Secondary Efficacy Outcomes:** For the responder rate (markedly effective, effective, ineffective based on PVC reduction categories: ≥90%, 50%–<90%, <50%), the total effective rate (markedly effective + effective) will be compared between groups using the Cochran-Mantel-Haenszel test stratified by center, or Fisher's exact test when appropriate. The 95% confidence interval for the difference in effective rates will also be calculated. For continuous secondary outcomes (PSQI score, SF-36 score, TCM syndrome total score, ECG indicators), similar MMRM or ANCOVA models will be applied as appropriate for each outcome.

All hypothesis tests will be two-sided with a significance level of α = 0.05. No interim analysis is planned. Statistical analyses will be performed using SAS version 9.4 or higher.

#### 2.10.4 Safety analysis

Adverse events will be evaluated using NCI CTCAE version 5.0. Analysis of adverse events will be based on the SAS. Data analysis mainly includes the frequency of adverse events, description of adverse events, adverse events related to the investigational drug, occurrence of serious adverse events, calculating the number of cases and incidence rates. Lists of all adverse events, adverse events related to the investigational drug, adverse events leading to withdrawal, and serious adverse events will be provided.

### 2.11 Data management and quality control

#### 2.11.1 Electronic case report form (eCRF) data entry and collection

All project-related data will be categorized, organized, and uploaded to the clinical Electronic Data Capture (EDC) system in a timely manner. Medical records, as the original documents of the clinical trial, should be preserved intact. Data in the research medical records should come from and be consistent with the source documents. Any observations and examination results during the trial should be recorded promptly, accurately, completely, standardizedly, and truthfully in the medical records and correctly filled into the research medical records. Changes should not be made arbitrarily. If correction is necessary due to an error, the original record must remain legible, and the correction should be signed and dated by the corrector. All laboratory data in the clinical trial should be recorded, and the original reports or their copies should be kept together with the research medical records. Data within the normal range should also be specifically recorded. Data significantly deviating from or outside the clinically acceptable range must be verified. Test items must specify the units of measurement used.

#### 2.11.2 Data management

All final patient data collected according to the protocol, including electronic Case Report Forms (eCRFs) and external data. Standard procedures will be followed to support accurate data collection. Data will be checked for outliers, logic, inconsistencies, and completeness. Review of medical records will be conducted in a manner that ensures patient confidentiality. The completeness and clarity of eCRFs need to be checked and cross-verified against source documents to monitor study progress. Audits also require direct access to source data, conducted with due consideration for data protection and medical confidentiality.

#### 2.11.3 Quality control

The investigator bears ultimate responsibility for all clinical data collected and reported entered into the eCRF. The investigator must provide an electronic signature in the EDC system to certify its accuracy, authenticity, and completeness. This project plans to conduct monitoring visits at each center.

## 3 Discussion

This study aims to systematically evaluate the efficacy and safety of Guipi Mixture combined with metoprolol tartrate tablets in treating PVCs (heart-spleen deficiency syndrome) through a randomized, double-blind, placebo-controlled, multicenter clinical trial. Current invasive treatment (catheter ablation) has strict indications (21), so most clinical guidelines recommend (3) β-blockers as initial therapy for frequent PVCs with persistent symptoms due to their tolerable safety and efficacy in treating ventricular arrhythmias and reducing the risk of sudden cardiac death (SCD). However, drug therapy has limitations such as a single target of action, risk of cardiovascular function impairment with long-term use, and limited improvement in patients’ quality of life. Therefore, this study aims to explore whether complementary and alternative medical strategies integrating TCM with Western medicine can achieve a more optimized risk-benefit balance, ensuring antiarrhythmic efficacy while improving drug safety profiles, ultimately translating into patient-centered health outcomes, including significant improvements in quality of life.

TCM theory posits that heart-spleen deficiency syndrome is one of the core pathogeneses of PVCs (22). Guipi Mixture, by tonifying Qi, fortifying the spleen, nourishing blood, and calming the spirit, is expected to regulate autonomic nerve function and improve myocardial energy metabolism through multiple targets (23), synergizing with metoprolol. If this study confirms the clinical value of Guipi Mixture, it will provide high-quality evidence-based support for integrated Chinese and Western medicine in treating arrhythmias and promote the optimization of individualized treatment strategies.

As the first rigorous randomized controlled trial targeting Guipi Mixture for PVCs, it employs a double-blind, placebo-controlled design and a multicenter collaboration model to minimize bias to the greatest extent. If the results are positive, they can provide direct evidence for the writing and updating of future guidelines and consensus, promote the inclusion of Chinese patent medicines in clinical pathways, and fill gaps in evidence-based research for TCM. Secondly, in addition to the primary endpoint (PVC count on 24-hour AECG), this study simultaneously evaluates TCM syndrome scores, PSQI sleep quality, and SF-36 quality of life scores, aligning with the holistic view of TCM “simultaneously treating the heart and spleen”. It is expected to reveal the unique advantages of Guipi Mixture in alleviating accompanying symptoms such as anxiety and insomnia, aiming to evaluate the impact of Guipi Mixture on the quality of life of PVC patients (heart-spleen deficiency syndrome), compensating for the insufficiency of previous drug therapies focused solely on heart rate control. Furthermore, this study adopts a multicenter clinical trial design, relying on 18 collaborating units covering six major geographical regions: North China, East China, Central China, South China, Southwest China, and Northwest China. This strategy, by including a geographically heterogeneous population, effectively controls potential selection bias inherent in single-center studies, enhances sample representativeness and result generalizability, and lays the foundation for the applicability of conclusions in different regional healthcare settings. Finally, the prespecified exploratory analyses (e.g., efficacy in different Lown grade subgroups, correlation between TCM syndromes and PVC count) will identify the population that benefits most, providing a basis for subsequent precise pattern differentiation-based medication and promoting the practical implementation of the “disease-syndrome combination” treatment model.

Guipi Mixture (containing Astragalus membranaceus, Codonopsis pilosula, Atractylodes macrocephala, Angelica sinensis, etc.) may exert synergistic antiarrhythmic effects through multiple pathways such as modulating autonomic nerve tone, inhibiting myocardial fibrosis, and improving myocardial microcirculation (24). Basic research indicates that astragaloside (25) can inhibit calcium overload in ventricular myocytes, and Angelica sinensis polysaccharide (26) has antioxidant stress effects, complementing the β-receptor blocking action of metoprolol. Although this study does not include mechanism exploratory endpoints, it can provide a clinical phenotype foundation for subsequent research, guiding the conduct of multi-omics analyses such as proteomics and metabolomics to deeply analyze the molecular network of “simultaneously treating the heart and spleen” in intervening arrhythmias, holding scientific significance for potential mechanisms.

However, the secondary endpoints in this study, such as TCM syndrome scores, PSQI, and SF-36 scales, rely on patient subjective reports and may be influenced by placebo effects or reporting bias. Although a double-blind design is used, results should be interpreted cautiously. Additionally, the treatment period is only 8 weeks, making it difficult to assess the long-term safety (e.g., cumulative liver and kidney damage) and sustained efficacy of Guipi Mixture. Future studies need to extend follow-up to 6–12 months to clarify long-term benefits.

Through rigorous methodological design, this study is expected to provide high-level clinical evidence for Guipi Mixture in treating PVCs of the heart-spleen deficiency type. If its efficacy-enhancing and toxicity-reducing effects are confirmed, it will promote the transition of integrated Chinese and Western medicine from “empirical medication” to “evidence-guided” practice, and lay the foundation for developing individualized TCM intervention plans and optimizing patient home management (such as lifestyle interventions and symptom monitoring). Ultimately, it aims to enhance the comprehensive management of arrhythmias and improve patients' quality of life.

## 4 Conclusion

This study is a randomized, double-blind, placebo-controlled, multi-center clinical trial designed to evaluate the efficacy and safety of Gui-Pi Mixture in treating premature ventricular contractions with the syndrome of deficiency of both the heart and spleen. Simultaneously, it aims to validate the impact of Gui-Pi Mixture on the quality of life of PVC patients presenting with this syndrome, thereby contributing therapeutic strategies for the integrated management of PVCs through Traditional Chinese Medicine and Western medicine approaches.
